# The mineral manaksite, KNaMnSi_4_O_10_, as a supercapattery-type electrochemical energy storage material†

**DOI:** 10.1039/d3ra03629d

**Published:** 2023-09-06

**Authors:** Gregarious Muungani, Michael N. Pillay, Werner E. van Zyl

**Affiliations:** a School of Chemistry and Physics, University of KwaZulu-Natal Westville Campus Durban 4000 South Africa vanzylw@ukzn.ac.za +27 31 260 3199

## Abstract

The manaksite mineral KNaMnSi_4_O_10_ was synthesized and used to fabricate electrodes, which were investigated for electrochemical energy storage (EES) application using cyclic voltammetry (CV), galvanostatic charge and discharge (GCD), and electrochemical impedance spectroscopy (EIS). Optimum weight percentages (wt%) of electrode components were established as 10 wt% polytetrafluoroethylene (PTFE) binder, 15 wt% RuO_2_ and 5 wt% carbon black. RuO_2_ was added to improve electrical conductivity. A ratio of 13 : 3 for KNaMnSi_4_O_10_ : RuO_2_ was used in the fabrication of the electrode. A study of the suitable electrolyte and corresponding concentration to use was done using NaOH and KOH, both at concentrations of 1 M, 3 M and 6 M, with 3 M NaOH as the optimum electrolyte and concentration. The KNaMnSi_4_O_10_ yielded a specific capacity of 106 mA h g^−1^. An investigation into the energy storage mechanism from a plot of log *I*(*ν*) *vs.* log *ν*, where *I* is current and *ν* is the scan rate gave a *b* value parameter of 0.8; that is, in-between 0.5 obtained for a pure battery material and 1.0 for a pure capacitor material. Accordingly, KNaMnSi_4_O_10_ exhibited a battery-supercapacitor duality phenomenon consistent with supercapattery materials. The KNaMnSi_4_O_10_ electrochemical system involved both capacitive and diffusion-controlled processes and was found to have good cyclic stability. It is concluded that KNaMnSi_4_O_10_ is a potential electrochemical energy storage material.

## Abstract 2

The mineral KNaMnSi_4_O_10_ was used to form electrodes and investigated for electrochemical energy storage (EES) application using cyclic voltammetry (CV), galvanostatic charge and discharge (GCD), and electrochemical impedance spectroscopy (EIS).

## Graphical abstract

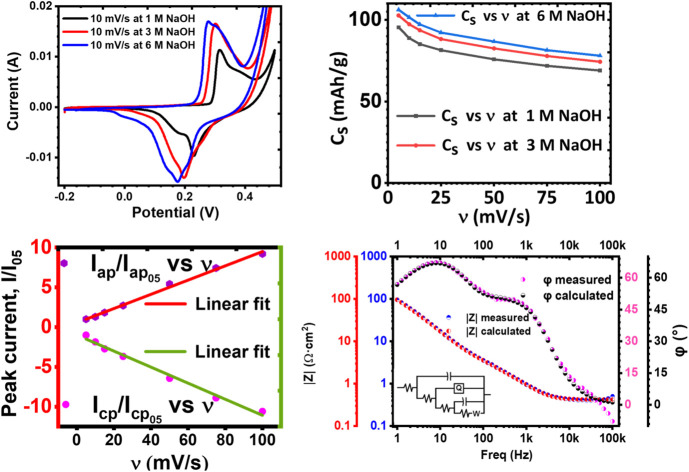

## 1 Introduction

The demand for sustainable energy solutions is on the rise due to increased energy consumption resulting from global population explosion, urbanization, industrialization, and advanced technological development. This occurs against the backdrop that continued overconsumption of fossil fuels as an unclean and limited resource facilitates its depletion. Clean and efficient energy storage materials were proposed to mitigate both energy demand and energy supply challenges.^1–4^ A prominent type of energy storage material is the electrical double layer capacitor (EDLC), which is a purely capacitive material as it stores charge electrostatically. Another type of material stores energy through redox processes. Each of these two types of energy storage materials has its pros and cons. The EDLC type of material has the advantage of high-power density, which results from fast charge storage and fast charge release since no phase transformations are involved such as those occurring through chemical reactions. On the other hand, the redox type energy storage material has a comparatively low-power density^5^ due to chemical reactions occurring in redox-based materials being slow compared to the electrostatic mechanism obtained in the EDLC. However, redox-type materials have a higher energy density than EDLC type materials. Besides these two traditional and opposed energy storage materials, a hybrid type material exists that exploits both the power capability of an EDLC and also the high energy storage capacity of a redox type material.^6,7^ This material has so-called supercapattery attributes^8,9^ and the need to investigate such innovative energy storage materials has emerged.^10–12^

Both organic and inorganic materials are being explored as potential electrochemical energy storage (EES) materials. Inorganic materials, in particular phyllosilicates, were explored both in supercapacitor application^13–17^ and battery applications.^18–24^ The choice for the use of phyllosilicates is attributed to their good mechanical and chemical properties, high specific surface area, cation exchange capacity, abundance, ready availability, cost-effectiveness, and eco-friendliness. In this work, KNaMnSi_4_O_10_ (manaksite), which is a member of the litidionite group comprising KNaCuSi_4_O_10_ (litidionite), KNaFeSi_4_O_10_ (fenaksite), KNaMnSi_4_O_10_ (manaksite)^25,26^ and KNa[Ca(H_2_O)][Si_4_O_10_] (calcinaksite),^27,28^ was synthesized and investigated for electrochemical energy storage application. The general formula of these litidionite group minerals is AA'M[Si_4_O_10_], where A and A′ are alkali cations (K^+^, Na^+^) and M is a bivalent cation (typically a transition-metal).^28^ The litidionite-group minerals have a heteropolyhedral framework of infinite silicate tubes [Si_8_O_20_]^8−^ with a hexagonal cross-section^28^ and they extend parallel to the *a* axis being connected by M_2_O_8_ dimeric units.^29^ The structure of these silicates is composed of continuous wall layers formed by edge-sharing octahedra and seven vertex polyhedra polyhedra^30^ with the tubular Si–O chains [Si_8_O_20_]^8−^ located between the walls. The presence of large cavities in tubes is generally occupied by large low-valent cations such as K^+^. These attributes are crucial in achieving desirable electrochemical energy storage performance.

On the other hand, certain transition metal oxides (TMOs), which are pseudocapacitive materials such as MnO_2_ and RuO_2_ were incorporated into electrochemical energy storage (EES) materials due to their variable oxidation states^31–33^ to improve the energy capability of the material through faradaic charge storage. In this work, ruthenia, RuO_2_, was added to KNaMnSi_4_O_10_ to improve electrical conductivity and the material was investigated for electrochemical energy storage application. Though RuO_2_ is a metal oxide, it has metallic-type conductivity that results from its Fermi level that is positioned in the middle of a conduction-band density-of-states curve.^34,35^ Furthermore, RuO_2_ is an n-type metal since electrons are the main charge carriers, unlike Ru which is a p-type metal.^36,37^ RuO_2_ has fast charge transfer, excellent reversible redox transitions,^38^ wide potential window (1.2 V), high proton conductivity,^35,39^ and has a high specific capacitance (900–1400 F g^−1^), with a theoretical capacitance of 2000 F g^−1^.^38,40,41^ RuO_2_ was extensively studied because of its ultra-large theoretical specific capacitance^40,42^ and high metallic electrical conductivity (10^5^ S cm^−1^), as well as excellent chemical stability at room temperature.^43–45^

In this work, KNaMnSi_4_O_10_ was synthesised hydrothermally and RuO_2_ was added to improve electrical conductivity. The KNaMnSi_4_O_10_ electrodes included 10 wt% polytetrafluoroethylene (PTFE) binder, 15 wt% RuO_2_ and 5 wt% carbon black. The KNaMnSi_4_O_10_ electrodes were investigated for electrochemical performance in 3 M NaOH and 3 M KOH electrolytes using a three-electrode configuration. This is the first-time electrochemical tests were conducted on KNaMnSi_4_O_10_. The KNaMnSi_4_O_10_ electrode exhibited a supercapattery phenomenon and the one electrochemically tested in NaOH had a relatively high specific capacity of 106 mA h g^−1^.

## 2 Experimental

### 2.1 Chemicals and materials

Sodium silicate solution (Na_2_O 8 wt%, SiO_2_ 27 wt%); KOH, KCl, MnSO_4_·4H_2_O, polytetrafluoroethylene (PTFE), 1-methyl-2-pyrrolidinone (NMP), carbon black, HCl, ethanol and Ni foam (150 mm × 150 mm) were purchased from Merck and used without further purification. Copper tape was purchased from BASi and used as received.

### 2.2 Characterisation methods

An X-ray diffractometer (Bruker AXS D8 Avance, Germany) fitted with a Cu Kα radiation source (wavelength = 0.154 nm) operating at 40 kV and 40 mA XRD was employed for powder X-ray analysis of the sample for 2*θ* values ranging from 5–90° at room temperature. Electrochemical studies were conducted using a Princeton Applied Research VersaSTAT 3 – Potentiostat Galvanostat. A three-electrode setup consisting of an Ag/AgCl electrode (reference electrode), working electrode (KNaMnSi_4_O_10_), and platinum wire (counter electrode) was utilized. The morphological information for KNaMnSi_4_O_10_ were established using a Transition Electron Microscopy (TEM) with a JEOL 1010 (Japan) Transmission Electron Microscope (TEM). Surface topography and composition of the samples were performed by a Zeiss Ultra Plus Field Emission Gun Scanning Electron Microscope (FEGSEM) equipped with an energy dispersive X-ray (EDX) detector (Germany). TEM imaging samples were prepared by adding them to ethanol in sonication tubes after which the sample was sonicated for 15 minutes. For SEM imaging, the samples were deposited each on a conductive carbon tape that was stuck to aluminum stubs. The samples were then coated three times with gold using a sputter coater to minimize charging during SEM imaging. The FTIR spectra of the samples were obtained using a Spectrum 100 infrared spectrometer equipped with a universal diamond crystal attenuated total reflection (ATR) accessory (PerkinElmer, USA) in the wavenumber range 380–4000 cm^−1^ at a resolution of 4 cm^−1^. The Raman spectra of the samples were obtained using a DeltaNu Advantage 532 high-performance Raman spectrometer fitted with a 532 nm solid-state frequency-doubled Nd:YAG laser that has a peak power of 200 mW and a 35 μm diameter focused beam. Its resolution ranges from 8–10 cm^−1^ and the spectral range from 200–3400 cm^−1^.

### 2.3 Synthesis of manaksite, KNaMnSi_4_O_10_

Manaksite was synthesized hydrothermally using a Teflon-lined autoclave according to literature.^25^ Briefly, two aqueous solutions were prepared (i) an alkaline solution comprising sodium silicate solution (sodium and silica source), KCl, KOH, and H_2_O and (ii) an aqueous solution of dissolved MnSO_4_·4H_2_O. The two solutions were added together and stirred to homogeneity to give a mixture of the following molar ratio 1MnO : 5.74SiO_2_ : 1.75Na_2_O : 5.87K_2_O : 355.32H_2_O. The homogenous mixture was transferred into a Teflon-lined autoclave and the reaction was carried out under static hydrothermal conditions at 230 °C for 7 days.

### 2.4 Fabrication of electrode and electrochemical measurements

Ni foam was used as a current collector on which the active material was loaded. First, the Ni foam (0.2 mm thick, 1 cm^2^ area) was cleaned by sonicating in 0.1 M HCl for 5 minutes, thrice rinsed with deionized water, sonicated in deionized water for 30 minutes, and then sonicated in ethanol for 1 h. The cleaned Ni foam was dried overnight in an oven at 110 °C after which it was loaded with the active material, KNaMNSi_4_O_10_. The KNaMnSi_4_O_10_ working electrode was fabricated in the ratio KNaMnSi_4_O_10_ : RuO_2_ : PTFE : CB as 36.5 : 7.5 : 5 : 1. The components were mixed to homogeneity after which 1-methyl-2-pyrrolidinone (NMP) was added to make a slurry. The slurry was sonicated for 1 hour and then coated on one side of a pre-treated Ni foam, as above. The coated Ni foam was then dried overnight in an oven at 110 °C and then densified at a pressure of 0.4 MPa. Cyclic voltammetry (CV), galvanostatic charge and discharge (GCD), and electrochemical impedance spectroscopy (EIS) measurements were carried out using a conventional three-electrode configuration. The nickel foam coated with KNaMnSi_4_O_10_ as the working electrode, the platinum electrode as the counter electrode and the Ag/AgCl (1.0 M KCl) electrode served as a reference electrode. Optimization experiments of the weight percentages (wt%) of the electrode components, in particular, the PTFE binder, RuO_2_ and the carbon black conductive additive were done using cyclic CV, EIS, and GCD. Experiments to determine the electrolyte to use between NaOH and KOH and what concentration (1 M, 3 M or 6 M) were performed. The AC EIS measurements were performed between 1 Hz and 100 kHz.

### 2.5 Electrochemical equations

The following equations were used to calculate specific parameters. The Scherrer equation, eqn (1)^46,47^

1 *D*_*hkl*_ = *Kλ*/(*B*_*hkl*_ cos *θ*)

where *D*_*hkl*_ is the crystallite size in the direction perpendicular to lattice planes, *hkl* are the Miller indices of planes being analyzed, *K* is a numerical factor related to crystalline shape, size, and width,^48^*λ* is the wavelength of X-rays, is the full width at half maximum of the X-ray peak in radians and *θ* is the Bragg angle.

The specific capacitance from the cyclic voltammogram, 
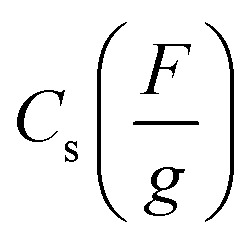
, was calculated by using the integrated voltammetric charge according to eqn (2)^47^2
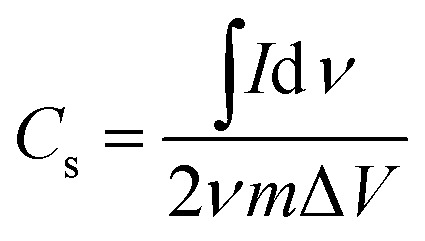
where *I*, *ν*, Δ*V* and *m* are the response current, scan rate (V s^−1^), and mass of active material loaded on the working electrode, respectively. Aside from the mass of the active material, current is influenced by the conductivity and concentration of the electrolyte, which is concerned with charge density.

Specific capacitance from the GCD was calculated using eqn (3)^20^3
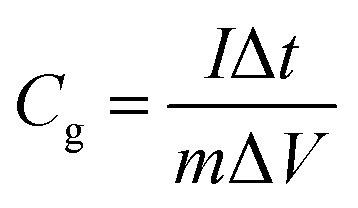
where *I* (in A) is the discharge current, *m* (g) is the total mass of the active materials, *t* (s) is the discharge time, *V* (V) is the potential during the discharge process after the IR drop; d*V*/d*t* is the slope of the discharge curve.

## 3 Results and discussion

### 3.1 Physical characterization

**Fig. 1:**
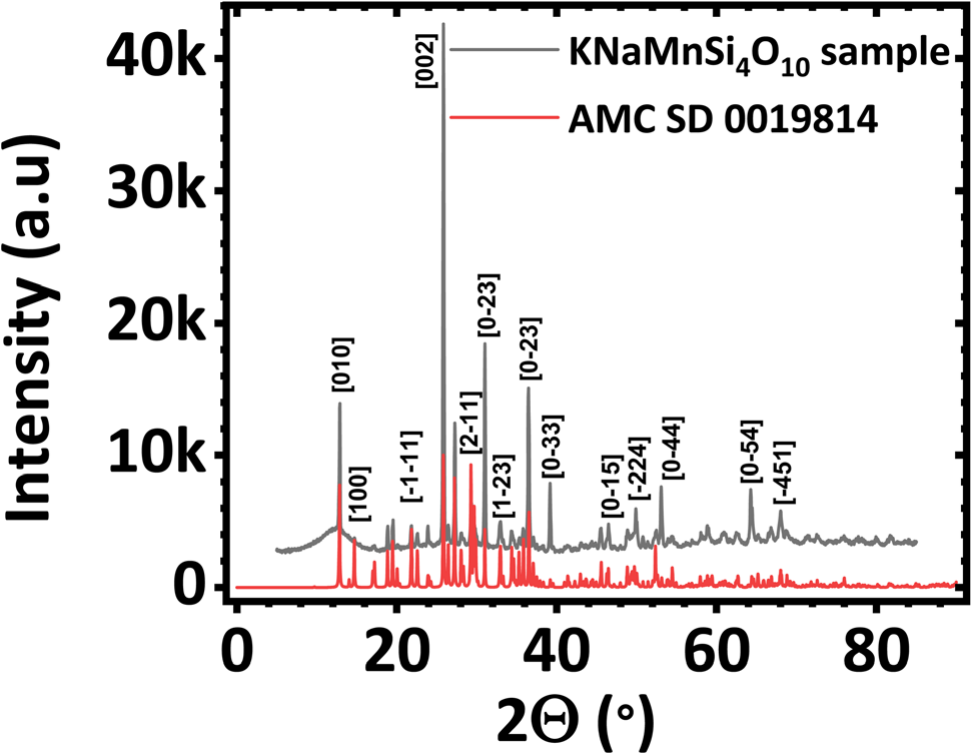
Powder X-ray diffractogram (PXRD) of (a) KNaMnSi_4_O_10_ sample compared against (b) KNaMnSi_4_O_10_ standard (AMCSD 0019814).

The PXRD diffractogram of the synthesized KNaMnSi_4_O_10_ sample is shown in Fig. 1(a) and compared against a standard AMCSD 0019814. The diffractogram obtained matches the American Mineralogist Crystal Structure Database reference standard, AMCSD 0019814 for KNaMnSi_4_O_10_ confirming successful synthesis. The sharp peaks of the PXRD diffractograms indicate that the material is crystalline, the crystallite size was calculated from the Scherrer equation using the diffraction data for the most intense peak and was found to be 47.40 nm. The full width at half maximum (FWHM) was first determined and was obtained as 2.655 × 10^−3^ radians denoting the formation of nano-crystallite size KNaMnSi_4_O_10_, which has the potential to increase the surface area exposed for electrostatic and electrochemical energy storage. The PXRD diffractogram of the RuO_2_ that was added to the KNaMnSi_4_O_10_ material to improve electrical conductivity is shown in Fig. S1.†

**Fig. 2:**
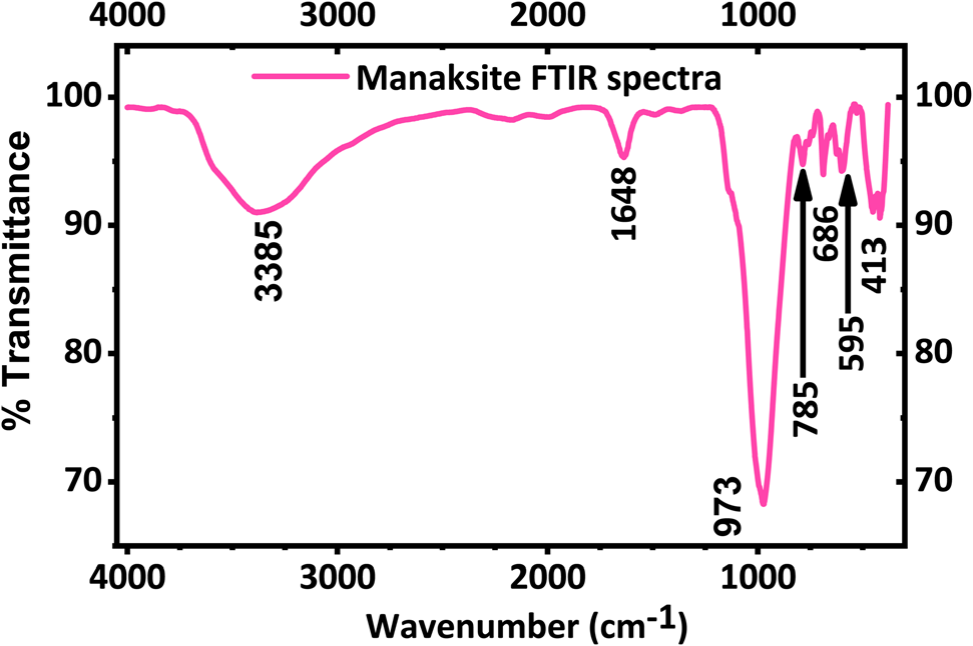
FT-IR spectra of KNaMnSi_4_O_10_ sample.

The FT-IR spectrum of KNaMnSi_4_O_10_ is shown in Fig. 2. A strong peak appears at 973 cm^−1^ in agreement with the literature^49^ and is a result of Si–O asymmetric stretching. Bands at 785, 686 and 595 cm^−1^ are due to Si–O bending vibrations of the tubular silicate radical Si_4_O_10_. The peaks at 452 and 416 cm^−1^ are due to the Na–O and K–O stretching vibrations, and Si–O bending vibrations. The band at 1638 cm^−1^ corresponds to H–O–H bending vibrations, as also observed for calcinaksite.^28^ The bands at 3340 and 3540 cm^−1^ correspond to weaker hydrogen bonds and, consequently, to longer O⋯O distances. Manaksite and fenaksite are regarded as anhydrous minerals, but the appearance of very weak water bands in their spectra shows the presence of minute amounts of water.^27^ It is also noted that KNaMnSi_4_O_10_ has some traces of water as shown by the peak at 1648 cm^−1^ and a broad peak around 3385 cm^−1^ even though the mineral is considered anhydrous. This research suggests that KNaMnSi_4_O_10_ contains traces of water, and this finding confirms other reports that KNaMnSi_4_O_10_ contains traces of water.^27,28,49^

**Fig. 3:**
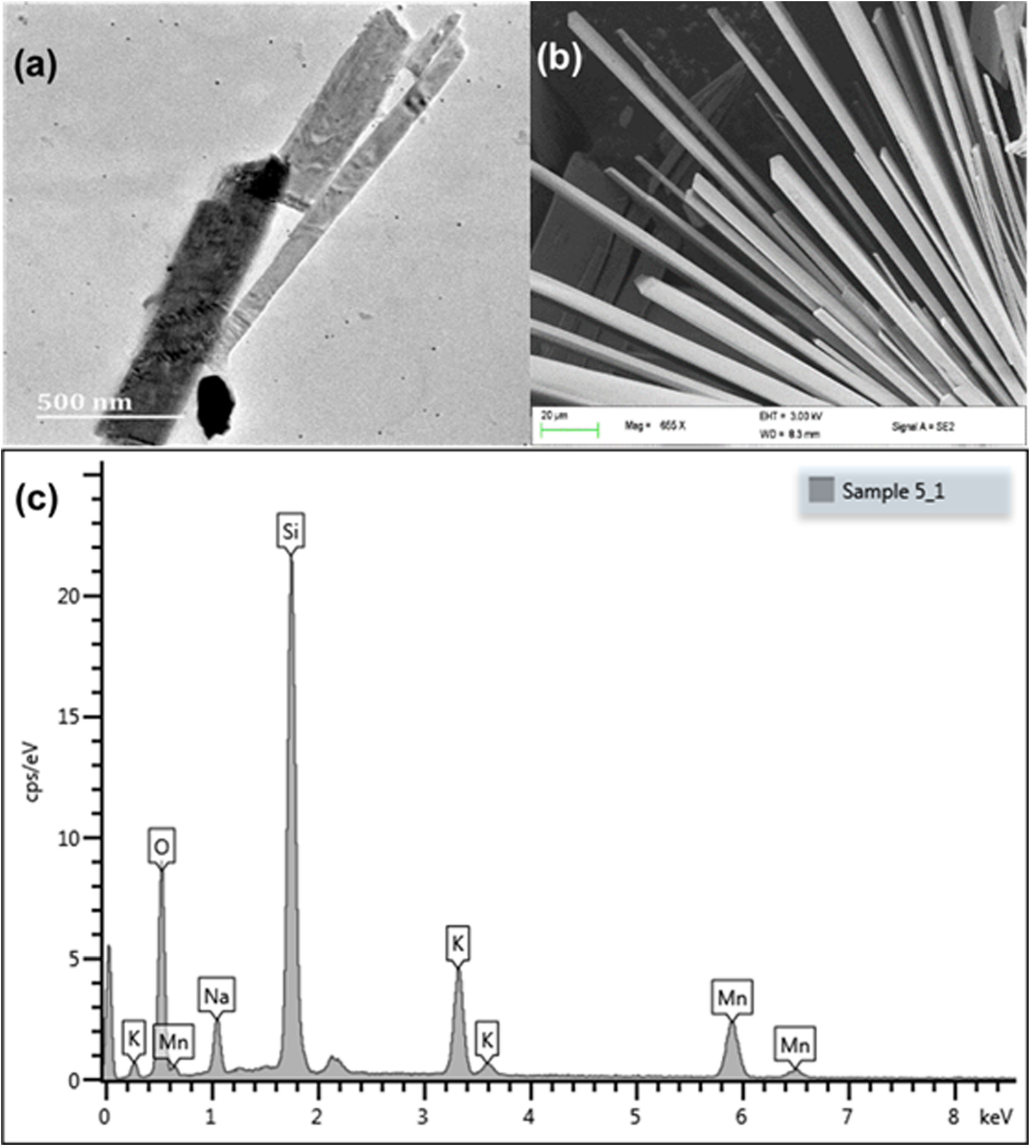
(a) TEM micrograph, (b) SEM and (c) SEM-EDX spectrum of KNaMnSi_4_O_10_.

Tubes of different sizes can be seen in the TEM image, Fig. 3(a). The SEM analysis showed that KNaMnSi_4_O_10_ was both layered and tubular, Fig. 3(b). The SEM image clearly showed the hexagonal cross-section of the tubes,^28,50^ which was attributed to the linking of the Si-tetrahedra through common vertices. The SEM-EDX showed the constituent elements of KNaMnSi_4_O_10_, which showed the material was pure and without contamination, Fig. 3(c). The finding corroborated the X-ray diffractogram, which only showed the presence of a single phase that matched the pattern for KNaMnSi_4_O_10_ in the database. Furthermore, the elements showed up as expected from their characteristic X-rays, which are at 3.312, 1.041, 5.894, 1.739, and 0.525 keV for K, Na, Mn, Si and O, respectively, for their spectral peak Kα and 0.637 keV for Mn Lα.^51^ The peak at 0 keV is a noise peak associated with the electronics of the detector and is stable with the temperature of the detector. The SEM and TEM images of the RuO_2_ that was added to the KNaMnSi_4_O_10_ material to improve electrical conductivity are shown in Fig S2 and S3,† respectively.

### 3.2 Optimization of electrode components

**Fig. 4:**
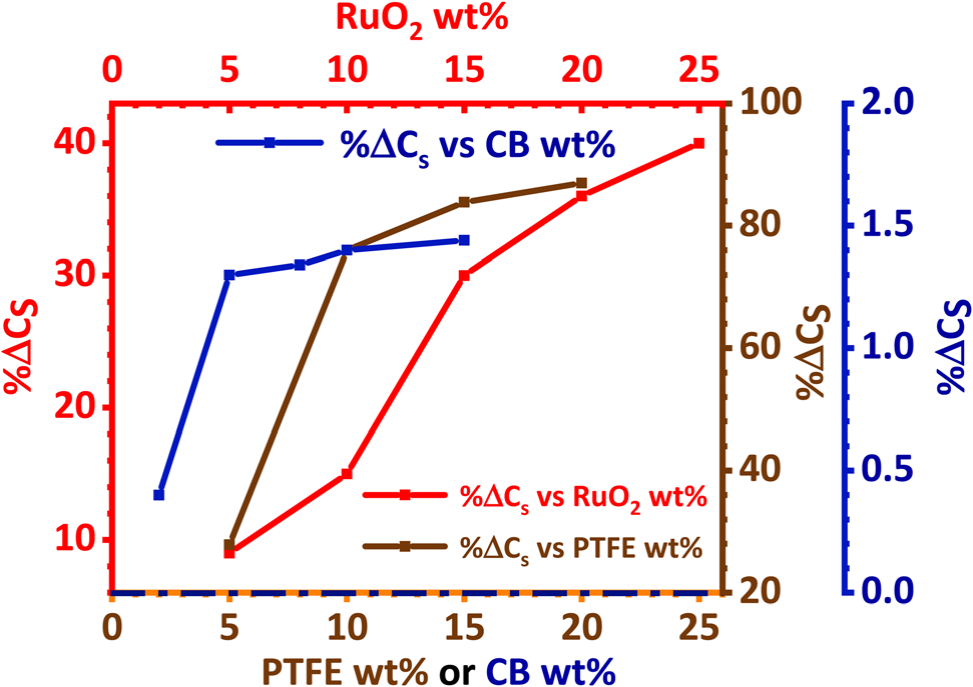
Percentage change of specific capacity (% Δ*C*_s_) with wt% variation of PTFE, RuO_2_ and carbon black.

The effect of RuO_2_, PTFE and carbon black on the specific capacity (*C*_s_) of the electrode was investigated, and it was found that 10 wt% PTFE binder was optimum. It was also observed that the addition of RuO_2_ to KNaMnSi_4_O_10_ improved the electrochemical energy storage performance of the material. The increase in *C*_s_ of KNaMnSi_4_O_10_ with the addition of RuO_2_ was a result of both its electrical conductivity and high capacitance whose theoretical value is 2000 F g^−1^ (ref. 40 and 52) and practically around 1400 F g^−1^.^39^ Specifically, 15 wt% RuO_2_ had a significant effect on the capacitance of the electrode. The results on the effect of the carbon black (CB) conductive additive on the performance of the electrode are shown in Fig. 4. It was established that 5 wt% CB was optimum, and such a low weight percentage can be attributed to the presence of RuO_2_, which has metallic-type bonding^35^ and exhibits metallic conductivity.^43,44^

### 3.3 Effect of electrolyte

**Fig. 5:**
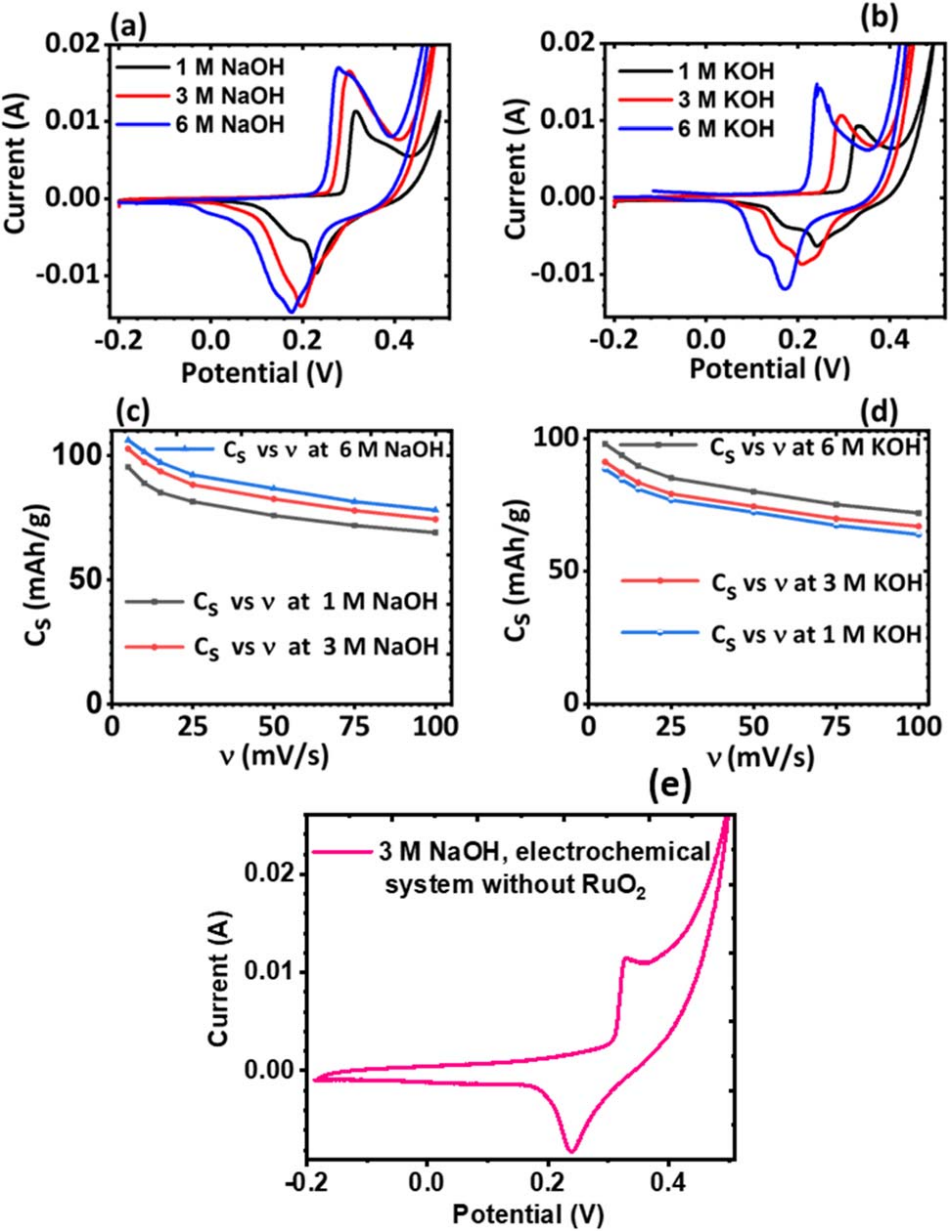
Plots of current *vs.* potential of KNaMnSi_4_O_10_ in varying concentrations of (a) NaOH electrolyte and (b) KOH electrolyte concentrations at 5 mV s^−1^; and plots of *C*_s_*vs. ν* for KNaMnSi_4_O_10_ in varying concentrations of (c) NaOH electrolyte, (d) KOH electrolyte and (e) cyclic voltammogram of the KNaMnSi_4_O_10_ without RuO_2_ electrochemical system in 3 M NaOH.

The effect of the electrolyte and scan rates on the current response and the specific capacity (*C*_s_) of KNaMnSi_4_O_10_ was investigated and results are presented in Fig. 5. It was observed that for NaOH electrolytes of concentrations 1 M, 3 M and 6 M (Fig. 5(c)), the 3 M NaOH electrolyte was the optimum concentration. This allowed for a satisfactorily performing and cost-effective electrochemical system for electrochemical energy storage, Fig. 5(a). On the other hand, for the KOH electrolytes, comparing the peak currents obtained and the specific capacity associated with the 1 M, 3 M and 6 M KOH (Fig. 5(b) and (d)), the 6 M KOH electrolyte was the optimum electrolyte, Fig. 5(b). Furthermore, it was established that the specific capacity obtained for the 3 M NaOH, which is 106 mA h g^−1^, was insignificantly less than 106.4 mA h g^−1^ obtained for the 6 M NaOH electrochemical system. Thus, considering that by comparison 3 NaOH is more economically viable than 6 M NaOH, the 3 M NaOH electrolyte was considered optimum and this solution was therefore used as the electrolyte in subsequent experiments. In addition, the specific capacity obtained for the 3 M NaOH at 106 mA h g^−1^, was higher than obtained for 6 M KOH at 99.6 mA h g^−1^ in the electrochemical system. It was expected, however, that the KOH electrochemical system would give a higher specific capacitance than the NaOH electrochemical system owing to the smaller hydration radius of K^+^ at 2.32 Å, relative to Na^+^ at 2.76 Å, which should be favourable to a significant degree of electrosorption and increased charge density. It is plausible that the large hydration radius of Na^+^ could create a larger electrostatic charge area that contributed to increased charge storage.

It was also noted that there was a slight current peak shift to less positive values, Fig. 5(a) and (b). This may have been a result of pH variations due to electrochemical reactions involving charge storage as proposed for possible reactions occurring in the system in Section 3.4.1. It was observed that the specific capacity decreases with an increase in scan rate though at a gradual rate, see Fig. 5(c) and (d). The cyclic voltammogram of the KNaMnSi_4_O_10_ without RuO_2_ electrochemical system in 3 M NaOH is shown in Fig. 5(e) and has an anodic current of comparatively small peak height compared to the KNaMnSi_4_O_10_ electrochemical system where RuO_2_ was added to improve conductivity. The capacitance for the KNaMnSi_4_O_10_ without RuO_2_ electrochemical system in 3 M NaOH was 71 mA h g^−1^ at 5 mV s^−1^ compared to 106 mA h g^−1^ obtained for the KNaMnSi_4_O_10_ with RuO_2_ electrochemical system. Therefore, despite RuO_2_ improving conductivity of the KNaMnSi_4_O_10_ electrode, it also contributed towards increased capacitance of the electrochemical system.

### 3.4 Electrochemical analysis of the optimized electrode

#### 3.4.1 Electrode characterization using the power law formula, *i*(*ν*) = *aν*^*b*^

**Fig. 6:**
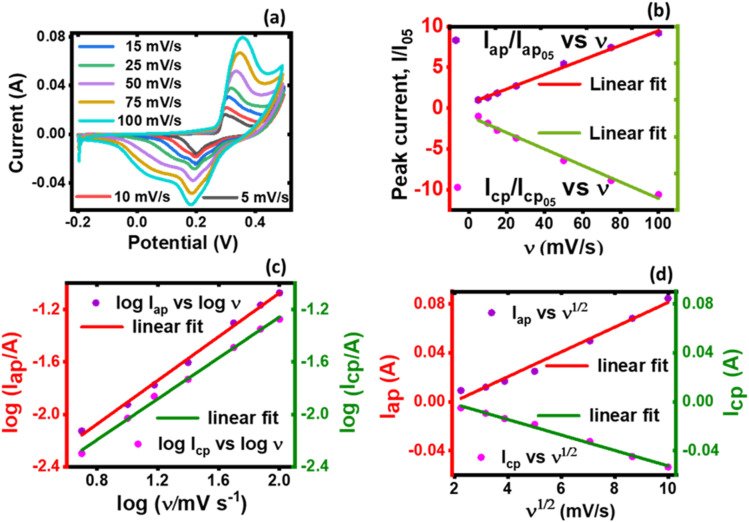
A plot of (a) current *vs.* potential from the CV, (b) peak current *vs.* normalized scan rate, (c) log peak current *vs.* log scan rate, and (d) log peak current *vs.* square-root of scan rate.

The electrochemical performance of the optimized electrode was investigated. Cyclic voltammetry was done and a plot of the current response to the varied scan rates was plotted, Fig. 6(a). It was observed that the peak current position shifts as the voltage sweep rate was increased in a manner that points to a quasi-reversible electrochemical system. This indicates that besides surface-based processes, the electrochemical system was characterized by other processes, possibly redox in nature.^53^ Thus, the presence of peaks suggests that the electrode material is not ideally capacitive and has some battery-type characteristic.^54^ From the cyclic voltammetry results, a plot of normalized anodic and cathodic peak currents against the scan rates; that is, 
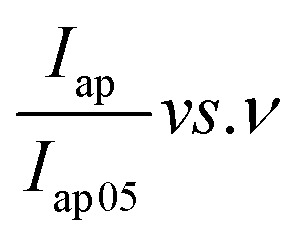
 and 
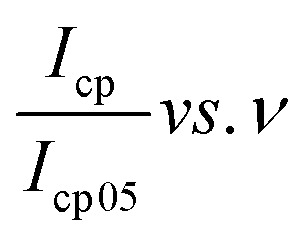
, was plotted, see Fig. 6(b). The current response was normalized against peak current obtained at a scan rate of 5 mV s^−1^. The current response obtained as a given scan rate was swept across a potential window can be disaggregated into a current that is generated from both the slow diffusion-controlled process (*i*_diff_); and either the charge that is needed to charge the double layer at the electrode–electrolyte interface, or charge that is needed to initiate faradaic reactions (*i*_cap_).^55,56^ The current response obeys the power law,^53,57^eqn (4):

4 *i*(*ν*) = *i*_cap_ + *i*_diff_ = *aν*^*b*^

which is rearranged as eqn (5):

5 log *i*(*ν*) = log *a* + *b* log *ν*

where both *a* and *b* are adjustable parameters. A plot of log *i*(*ν*) *vs.* log *ν*, which in this case is Fig. 6(c), is linear according to eqn (5). The slope of the plot gives the parameter *b* value, which elucidates the kinetics of the electrochemical reactions, classification of the material, and charge storage mechanism.^56^ A *b* value parameter of 1 is characteristic of capacitive or fast surface redox reactions^58^ whilst a *b* value parameter of 0.5 is characteristic of slow diffusion-limited faradaic processes. An electrochemical process yielding 0.5 < *b* < 1 represents a material or an electrochemical system of hybrid characteristics, that is both capacitive and diffusion-controlled processes are obtained to varying degrees. Such material or electrochemical system is said to have supercapattery attributes.

Both the normalized anodic and cathodic peak current responses to the scan rate behave linearly, see Fig. 6(b). Since these linear regression lines do not superimpose each other, it reveals that the redox process obtained in the electrochemical system is not perfectly reversible, although close. This observation has also manifested in the cyclic voltammograms, Fig. 5(a) and (b), where an observable difference in the shape of their current peak curves shows coupled with the notable variance of the magnitudes of the anodic and cathodic peak values, though small. In particular, the linear regression equation for the normalized anodic peak current (*I*_ap_/*I*_ap05_) *vs. ν* is *I*_ap_ = 0.553*A* + 0.0894*ν* where the slope is 0.0894, the intercept is 0.553, and the coefficient of correlation is 0.947. The linear regression equation of the normalized cathodic peak current *I*_cp_/*I*_cp05_*vs. ν* is *I*_cp_ = −0.989*A* − 0.1010*ν* where the slope is −0.1010, the intercept is −0.989, and the coefficient of correlation is 0.896. Notably, the two coefficients of correlation obtained for the anodic and cathodic peak current plots against scan rate, respectively, are high, though they exhibit some deviation from linearity. This observation implies that the parameter *b* value is not 1; if it was, then eqn (4) would be *i*(*ν*) = *aν* and *i*(*ν*) *vs. ν* would be perfectly linear. Consequently, further plots are needed to clarify the source of the current response obtained from the KNaMnSi_4_O_10_ electrochemical system as the voltage scan rate is swept across the potential window.

In this respect, a plot of the logarithm of the peak current, either anodic or cathodic, against the logarithm of the scan rate was performed, Fig. 6(c). As expected from the mathematical relationship, eqn (5), a linear relationship between both log *I*_ap_ and log *I*_cp_*vs.* log *ν* was obtained. A linear regression equation of the anodic processes from Fig. 6(c) was found to be log *I*_ap_ = −2.74 + 0.833 log *ν* where the intercept is −2.74, the slope is 0.833, and the linear coefficient of correlation is 0.9964. On the other hand, the linear regression equation for the cathodic processes, from Fig. 6(c), is log *I*_cp_ = −2.82 + 0.781 log *ν* where the slope is 0.781, the intercept is −2.82 and the coefficient of correlation is 0.9986. It should be noted that the slope of the log *I*_ap or cp_*vs.* log *ν* is the parameter *b* value. The |*b*| value for both anodic and cathodic reactions lie between 0.5 and 1.0. As explained above, such *b* values indicate that the material under investigation is characteristic of weighted capacitive or pseudocapacitive and battery-type electrochemical properties. The obtained |*b*| ≠ 1 reinforces the presence of peaks in the obtained cyclic voltammogram. Otherwise, if |*b*| = 1, which is ideal for capacitive double layer charging, implying that the electrochemical process is controlled by surface-dominant reactions, then a rectangular-shaped cyclic voltammogram would be expected. As alluded to earlier, the |*b*| value can be used to characterize materials as pseudocapacitive or battery-type materials or hybrid; that is, supercapattery type of material.

Since the electrochemical system also exhibits a semi-infinite diffusion process, a plot of *I*_ap_ and *I*_cp_*vs. ν*^1/2^ was done, Fig. 6(d). When semi-infinite diffusion dominates the electrochemical system, *I*_*p*_(*V*) ∝ *ν* (*i.e.*, d*i*/d*ν*^1/2^ = constant).^55,59^ The two processes can be expressed mathematically through the equation, eqn (6):

6 *i*(*V*) = *i*_cap_ + *i*_diff_ = *k*_1_*ν* + *k*_2_*ν*^1/2^

or, eqn (7):

7 *i*(*V*)/*ν*^1/2^ = *k*_1_*ν*^1/2^ + *k*_2_

The linear peak currents for both anodic and cathodic peak currents varied linearly with the square root of scan rate, that is *I*_ap or cp_ ∝ *ν*^1/2^ as shown in Fig. 6(d). The linear regression of the response of the anodic peak current to the scan rate that was swept across the voltage window is *I*_ap_ = −0.01984 + 0.0101*ν*^1/2^ where the intercept is −0.01984, the slope is 0.0101, and the coefficient of correlation *r* is 0.9821. Similarly, the linear regression for the response of the cathodic peak to scan is *I*_cp_ = 0.01078 + 0.00631*ν*^1/2^ where the intercept is, the slope is 0.00631 A V^1/2^ s^−1/2^ and the coefficient of correlation is 0.9930.

It was observed that the linear fit of both relations; that is, the anode and cathode peak currents to the square root of the scan rate shows a linear variation to a significant extent, which suggests that the electrode materials participated in redox reactions that were quasi-reversible and of a diffusion-controlled process.^60^ But a deviation is seen, especially with the peak anodic currents to the square root of the scan rate. This points to the existence of another source of current obtainable in the electrochemical environment that is different from the semi-infinite diffusion process, which *I*_ap or cp_ ∝ *ν*^1/2^ explores, and the presence of a capacitive charge process is observed. This is contributed to RuO_2_ which is known to have a pseudocapacitive behavior^61^ whilst KNaMnSi_4_O_10_ dominates the semi-infinite diffusion process. The current response varies with *ν*^1/2^ in the case of a reaction that is limited by semi-infinite linear diffusion whilst for a capacitive (surface-controlled) process, the current varies directly with *v*.

The anodic peak at 0.24 V corresponds to reaction,Ni + 2OH^−^ → Ni(OH)_2_ + 2e^−^,whilst the cathodic peak at 0.30 V is for reaction,Ni(OH)_2_ + OH^−^ ↔ NiOOH + H_2_O + e^−^.^62^

Nickel foam thus contributes to the specific capacitance of the electrode though to a small extent noting the small peak obtained for the CV of Ni foam and of KNaMnSi_4_O_10_ without RuO_2_ compared to that of KNaMnSi_4_O_10_ with RuO_2_. The capacitance of KNaMnSi_4_O_10_ with RuO_2_ arises from the pseudocapacitance contribution from RuO_2_ through a faradaic reaction,RuO_2_ + *δ*H^+^ + *δ*e^−^ ↔ RuO_2−*δ*_(OH)*δ* (1 ≥ *δ* ≥ 0).^63^

The contribution of RuO_2_ to the specific capacitance (*C*_s_) of 106 mA h g^−1^ for the KNaMnSi_4_O_10_ with RuO_2_ was found to be 32.5% suggesting that it contributed 34.45 mA h g^−1^.

#### 3.4.2 Galvanostatic charge and discharge (GCD) analysis

**Fig. 7:**
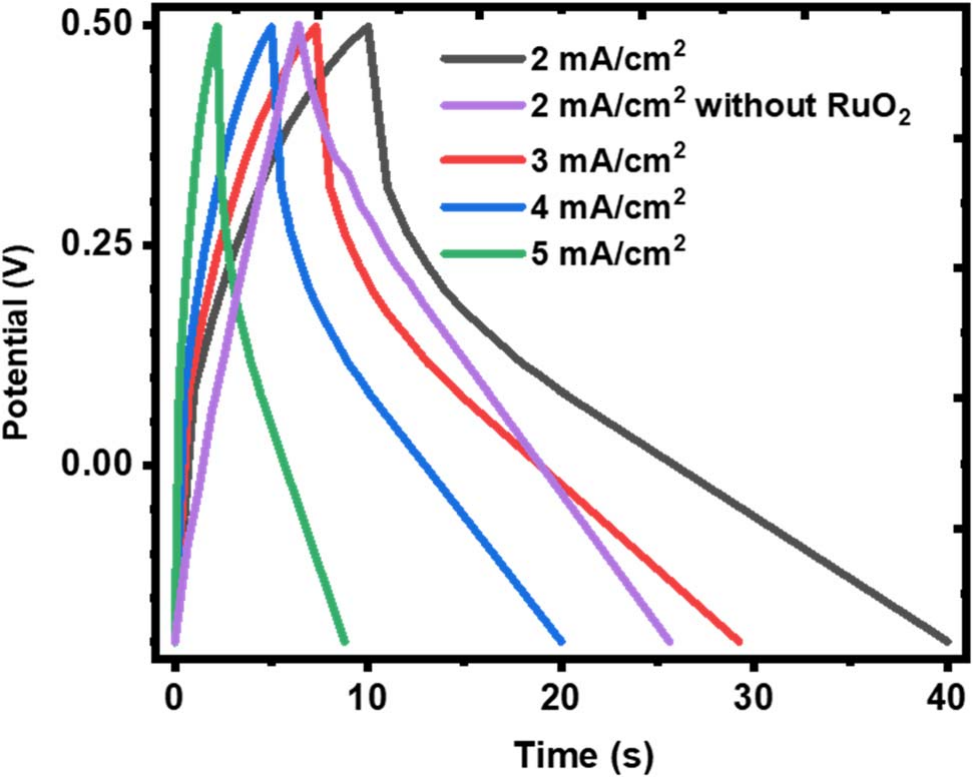
Charge and discharge curves of the KNaMnSi_4_O_10_ electrode at different current densities.

The KNaMnSi_4_O_10_ electrode was characterized using the galvanostatic charge and discharge technique and the results are plotted in Fig. 7. Consistent with the literature,^64^ specific capacity increased with decreasing current density, that is, from 5 mA cm^−2^ to 2 mA cm^−2^ and a specific capacity of 93 mA h g^−1^ was found at 2 mA cm^−2^. This specific capacity, 93 mA h g^−1^, is less than the 106 mA h g^−1^ obtained from the cyclic voltammetry technique. The lower specific capacity value obtained in GCD may be attributed to some of the charge stored during the charging stage may not be completely discharged, thereby reducing the amount of the charge available for discharge. Also, the obtained specific capacity depends on peculiarities of parameters that are identified with each instrument and the attended principles of instrumental operation. For CV, the scan rate is varied whilst for GCD, the current density is the crucial independent variable. In essence, the fundamental basis of operation of GCD and CV instrumentation and techniques are different, but the obtained specific capacity values should have some resemblance within the limit of their core principles of operation.

It is also observed that the charge and discharge curves have shapes that deviate from a near perfect triangle as expected of EDLC or pseudocapacitors with surface redox reactions but resembles a pseudocapacitor with intercalation^65^ and closely depicts supercapattery material.^8^ This shows that the KNaMnSi_4_O_10_ electrode is not purely capacitive since the charge and discharge segments of the plots deviate from linearity. This finding confirms the observation already established above from this work that the KNaMnSi_4_O_10_ electrode presents supercapattery attributes, which draw from those of pure capacitive materials and battery material giving it a battery-supercapacitor duality, indicating that the involvement of faradaic reactions is involved.^66^ Also seen from the charge and discharge curves is that the plots do not exhibit a notable *iR* drop at the apex at the onset of the discharge process. This reveals that the internal resistance of the KNaMnSi_4_O_10_ electrode is low, which is preferable. It is also observed that the GCD curves resemble more a capacitor on charging and a battery on discharging, which shows some slight variation with what is described for materials with a *b* value parameter between 0.5 and 1. This suggests that there may be subtle variations or sub-categories of materials that are grouped in the aforementioned *b* value parameter range and yet shows different electrochemical behaviour.

#### 3.4.3 Effect of *ν* on *C*_s_ and cyclic performance

**Fig. 8:**
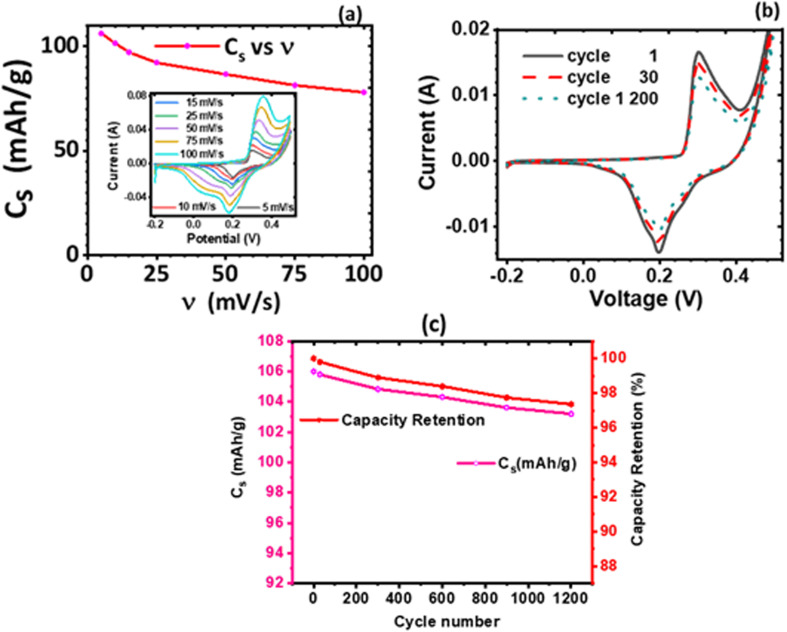
(a) A plot of *C*_s_*vs. ν* for KNaMnSi_4_O_10_ electrode in 3 M NaOH (insert: a plot of *I vs. V* at selected cycles – 5–100 mV s^−1^), (b) a plot of *I vs. V* at 5 mV s^−1^ for 1200 cycles, (c) showing capacity retention *vs.* cycle number and *C*_s_ (mA h g^−1^) *vs.* cycle number.

An investigation into the variation of *C*_s_ with *ν* was done and the results are shown in Fig. 8(a). The results showed that *C*_s_ increases with a decrease in *ν*, and *C*_s_ inversely varies with *ν*; that is, 
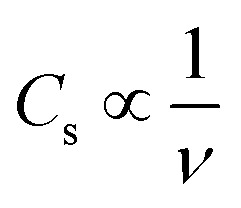
. Furthermore, an investigation of the cyclic performance of KNaMnSi_4_O_10_ electrode was carried out, and the results obtained are shown in Fig. 8(b). The current response of KNaMnSi_4_O_10_ electrode decreased to a trivial extent after 1200 cycles, Fig. 8(b), with a downward variation of 2.6% as illustrated on Fig. 8(c), which shows capacity retention *vs.* cycle number. The relatively small downward variation of specific capacitance as the cycle number increases demonstrated that the KNaMnSi_4_O_10_ electrode had an appreciable extent of cyclic stability; Fig. 8(c) also shows capacity retention *vs.* specific capacitance at 5 mV s^−1^. As confirmed by capacity retention, the KNaMnSi_4_O_10_ electrode has an appreciable cyclic stability.

#### 3.4.4 Electrochemical impedance spectroscopy (EIS) characterization

**Fig. 9:**
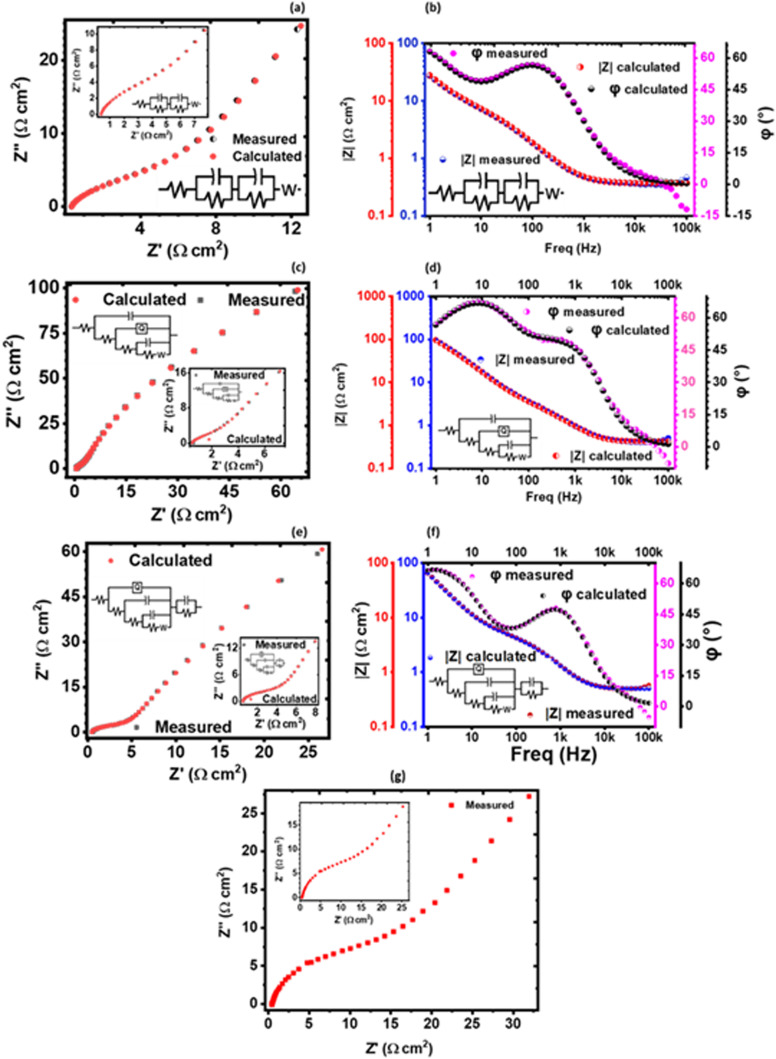
Nyquist plots (a), (c) and (e) and Bode plots (b), (d) and (f) for the KNaMnSi_4_O_10_ electrode in 1 M, 3 M and 6 M NaOH, respectively and (g) Bode plots for the KNaMnSi_4_O_10_ electrode without RuO_2_ in 3 M NaOH.

**Fig. 10:**
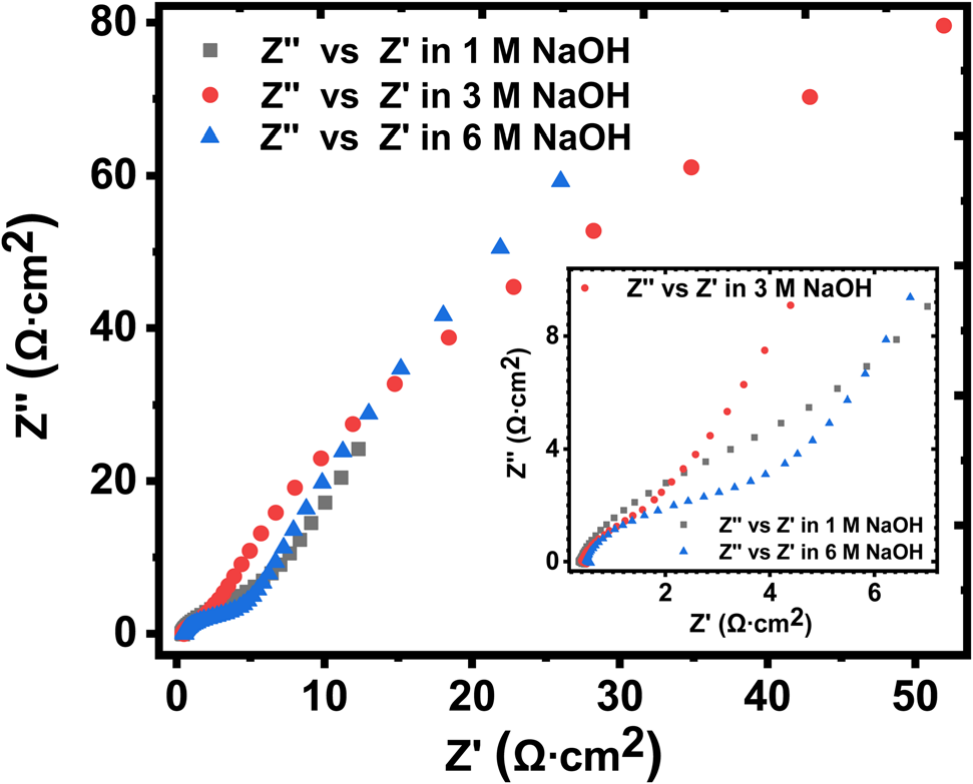
Nyquist plots for the KNaMnSi_4_O_10_ electrode in 1 M, 3 M and 6 M NaOH, respectively, drawn on the same axes for comparison.

Electrochemical impedance spectroscopy (EIS) of the KNaMnSi_4_O_10_ with RuO_2_ electrochemical system and of the KNaMnSi_4_O_10_ electrochemical system without RuO_2_ was done in the frequency range of 100 kHz to 1 Hz. The EIS results obtained are plotted in Fig. 9 and 10. A study of the plotted EIS results showed that they comprise high and low-frequency regions as is the case with such plots. The high-frequency region of the Nyquist plots, Fig. 9(a), (c), (e) and (g) showed the presence of semicircles to different degrees. The size of the semicircle provide insight into the magnitude of the charge-transfer resistance (*R*_CT_) – a large diameter of the semicircle implies a high *R*_CT_. In this work, the resistance emanates from the interface of the KNaMnSi_4_O_10_ electrode and the NaOH electrolyte. The charge transfer resistance (*R*_CT_), involves electrochemical reactions on the electrode surface^67^ and is determined from the diameter of the arc obtained at the high-frequency region. It was found that the *R*_CT_ for the KNaMnSi_4_O_10_ electrode with RuO_2_ in 1 M, 3 M, and 6 M NaOH was 5.401, 1.064, and 3.309 Ω cm^2^, respectively whilst that of KNaMnSi_4_O_10_ electrode without RuO_2_ in 3 M NaOH was 10.2 Ω cm^2^. The 3 M NaOH electrolyte gave the least *R*_CT_, suggesting that the electrochemical reactions that were involved in energy storage occurred with relative ease. The low *R*_CT_ obtained may be attributed to the presence of RuO_2_ in the KNaMnSi_4_O_10_ electrode that was added to improve electrical conductivity noting that R_CT_ of the KNaMnSi_4_O_10_ electrode without RuO_2_ was comparatively high. This finding reinforced the earlier result that 3 M NaOH electrolyte was optimum to use. On the other hand, the low-frequency region is characterized by a linear part. The more vertical the line, the closer the electrochemical system is to an ideal capacitor,^68^ a pure capacitor is characterized by a phase angle (*φ*) of 90°^69,70^ and low diffusion resistance of ions in the structure of the electrodes.^71^ For a pseudo-capacitor, *φ* < 90°.^68^

The solution resistance (RS), which is the non-zero intercept of the Nyquist plot to the Z′ axis; that is, the real impedance axis^72^ was determined to be 0.449, 0.496, and 0.571 Ω cm^2^ for 1 M, 3 M and 6 M NaOH electrolyte, respectively. In this work, the solution resistance comprises the internal resistance of the active material (the KNaMnSi_4_O_10_ electrode) and the ionic resistance of the electrolyte (NaOH), and the contact resistance obtained at the active material/current collector interface.^73–75^ The relatively low values obtained for both the RS and *R*_CT_ are attributed to the excellent conductivity of RuO_2_ and also the penetration ability of the electrolyte ions. A smaller the RS value implies close to perfect reversibility of the process taking place at the electrode/electrolyte solution interface.

The Warburg impedance (*W*), which is the slope of the line on the Nyquist plot that meets Z′ at 45° was also established. In particular, the Warburg resistance for the KNaMnSi_4_O_10_ electrode in 1 M, 3 M, and 6 M NaOH electrolytes was found to be 3.55 Ω cm^2^, 1.43 Ω cm^2^ and 3.59 Ω cm^2^ as shown in Fig. 10 where all the plots are presented on the same graph for comparison. The Warburg impedance is a parameter that demarcates the transition from high to low frequency^76^ and results from the frequency dependence of ion diffusion/transport in the electrolyte to the electrode surface.^77^ It was observed that the Warburg impedance obtained in for the 3 M NaOH electrochemical system was lower than the others, and confirmed the initial finding that the 3 M NaOH electrolyte was the optimum. Also noted from the Bode plots, Fig. 9(b), (d) and (f) was that the impedance of the KNaMnSi_4_O_10_ electrode in 1 M, 3 M, and 6 M NaOH was similar at high-frequency region.

A plot of phase angle, *φ*, as a function of frequency, the Bode plot was also done to further characterize the KNaMnSi_4_O_10_ electrode in terms of the charge storage mechanism. It was established that the KNaMnSi_4_O_10_ electrode had a phase angle of 57°, 67.5° and 48° in 1 M, 3 M and 6 M NaOH electrochemical system, respectively. The 3 M NaOH has the highest phase angle, which value is set at about 3/4 of an ideal capacitor whose phase angle is 90°. This finding corroborates the results from the log *I vs.* log *ν* plot, Fig. 6(b), that showed the KNaMnSi_4_O_10_ electrode is not purely capacitive but also exhibits some redox properties, which affords its supercapattery properties. It was also observed, Fig. 9(d), that the modelled equivalence circuit for the KNaMnSi_4_O_10_ electrode in the 1 M, 3 M, and 6 M NaOH electrolyte had a close match to the measured plot.

The obtained EIS results that gave the Nyquist and Bode plots were modelled to give equivalent model circuits (ECs) for the KNaMnSi_4_O_10_ electrochemical system in 1 M, 3 M, and 6 M NaOH electrolytes as shown in Fig. 9. It was observed that all the circuits show Warburg resistance, whose implications were aforementioned. The modelled equivalent circuits were appreciably close to the measured value with most parameters having a standard deviation (*σ*) from the measured values that were <10%. In particular, the KNaMnSi_4_O_10_ electrode in 3 M NaOH, which was the optimum electrochemical system, had an equivalence circuit involving a constant phase element (CPE). A CPE originates from internal factors such as intercalation/deintercalation, active diffusion, homogeneity disorder in an electrode–electrolyte interface, relaxation time distribution, and most decisively nature and porosity of the as-synthesized electrode.^78^

## 4 Conclusions

KNaMnSi_4_O_10_ was synthesized by a hydrothermal method. The NaOH electrolyte performed comparatively better than KOH in the application of the KNaMnSi_4_O_10_ electrode for electrochemical energy storage. For the 3 M NaOH and the 6 M KOH electrolytes, the specific capacity for the two electrochemical systems was almost the same, but with cost considerations, the 3 M NaOH electrolyte was found to be the optimum and more cost-effective electrolyte. The optimum weight percentages (wt%) for the electrode components were found to be 10 wt% PTFE binder, 5 wt% carbon black conductive additive, and 15 wt% for RuO_2_. The KNaMnSi_4_O_10_ electrode in 3 M NaOH electrolyte gave a specific capacity of 106 mA h g^−1^. Plots from the power law formula revealed that the KNaMnSi_4_O_10_ electrode had a parameter *b* value of 0.833 and 0.781 when characterized using the anodic and cathodic electrochemical process, respectively. Noteworthy is that both anodic and cathodic processes derived parameter *b* value lies between 0.5 and 1, that is 0.5 < *b* < 1. This indicated that the KNaMnSi_4_O_10_ electrode had properties that encompass both capacitive materials (*b* = 1) and battery-type materials (*b* = 0.5), *i.e.* a battery-supercapacitor duality phenomenon. Hence, the KNaMnSi_4_O_10_ electrochemical system is comprised of both capacitive charging and diffusion-limited processes, which gives the KNaMnSi_4_O_10_ electrode supercapattery attributes. Further investigations on different types of electrolyte and transition-metal oxides on the specific capacitance of KNaMnSi_4_O_10_ and other members of the litidionite series is required to further leverage better fabrication of electrochemical storage materials.

## Author contributions

Gregarious Muungani: conceptualization, data analysis, methodology, experimental investigation, writing first draft; Michael N. Pillay: conceptualization, data collection; Werner E. van Zyl: conceptualization, supervision, research funding, final editing.

## Conflicts of interest

The authors declare no conflict of interest.

## Supplementary Material

RA-013-D3RA03629D-s001
