# Supplementary material for: The mineral manaksite, KNaMnSi4O10, as a supercapattery-type electrochemical energy storage material
Source: RSC Adv. 2023 Sep 6;13(38):26732–43. doi: 10.1039/d3ra03629d (PMC10481385; doi:10.1039/d3ra03629d)
Supplement: RA-013-D3RA03629D-s001 [file RA-013-D3RA03629D-s001.pdf]

Electronic Supplementary Material (ESI) for RSC Advances.
This journal is © The Royal Society of Chemistry 2023

ELECTRONIC SUPPLEMENTARY INFORMATION

The mineral manaksite, $KNaMnSi_4O_{10}$, as a supercapattery-type electrochemical energy storage material

Gregarious Muungani, Michael N. Pillay, Werner E. van Zyl*

School of Chemistry and Physics, University of KwaZulu-Natal, Westville Campus, Durban, 4000, South Africa

Email: vanzylw@ukzn.ac.za

PXRD analysis of RuO_2

The PXRD defractogram is shown and the (h,k,l) indices of respective peaks are indicated.

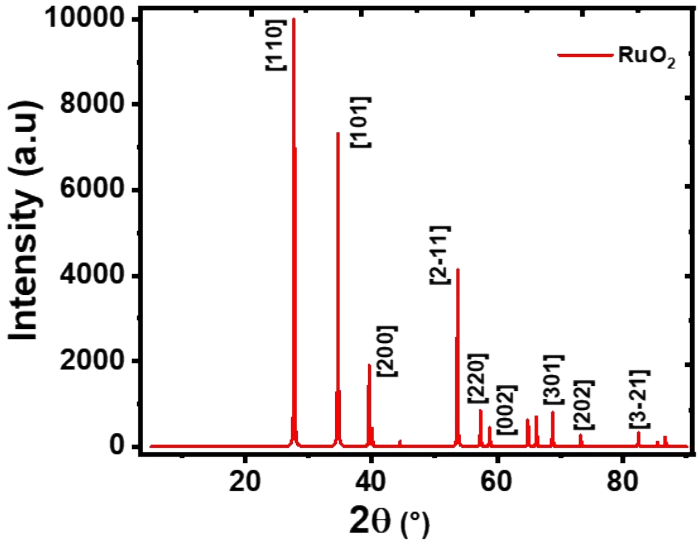

Fig. S1. Powder X-ray diffractogram (PXRD) of RuO_2 sample.

SEM and TEM analysis

SEM and TEM analysis of RuO_2 were performed and the images are shown in Fig. S2 and Fig. S3.

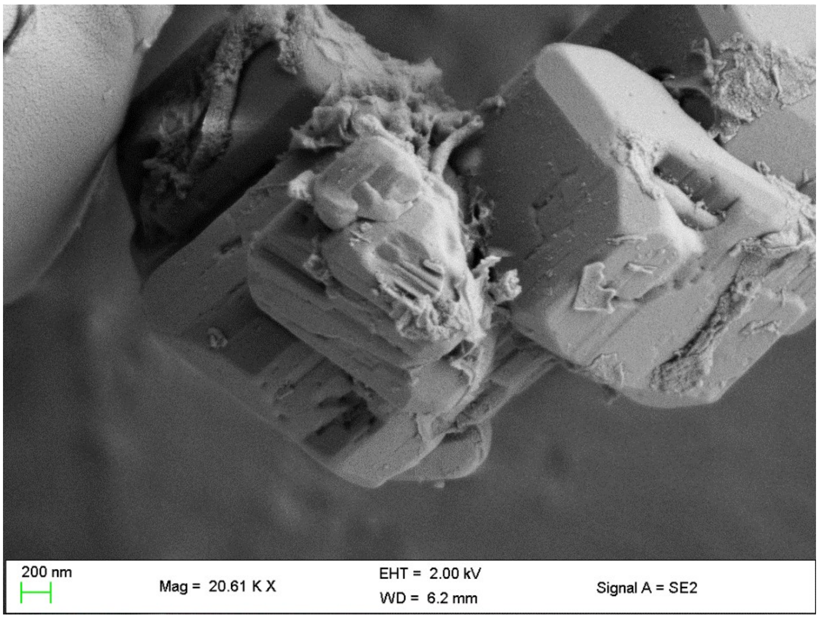

Fig. S2. (a) TEM micrograph, (b) SEM and (c) SEM-EDX spectrum of RuO_2.

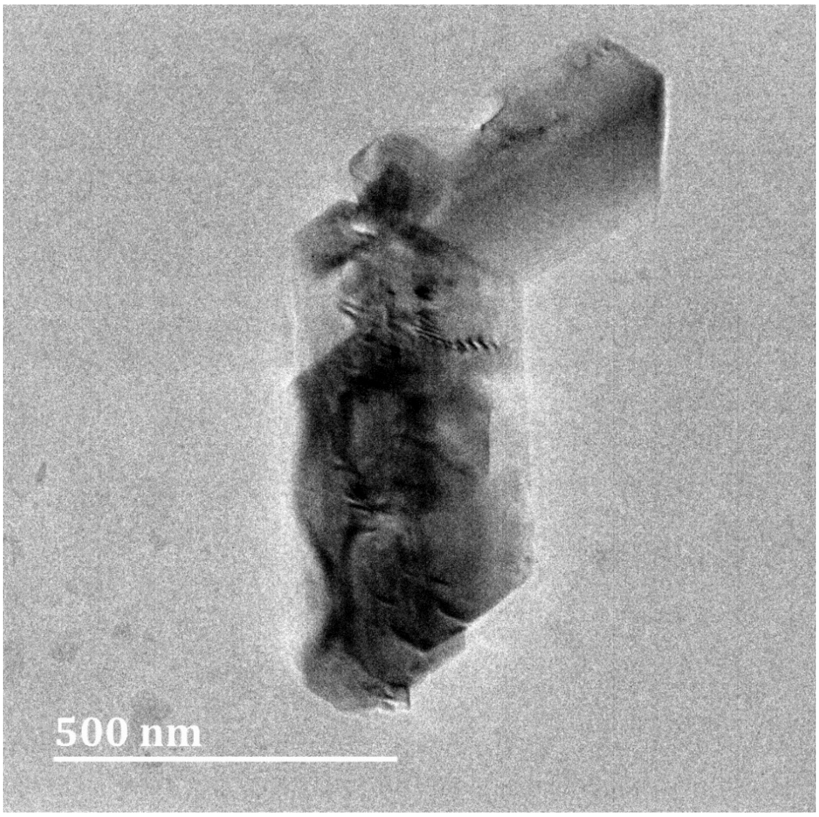

Fig. S3. (a) TEM micrograph of RuO_2.
